# Terahertz near-field microscopy of metallic circular split ring resonators with graphene in the gap

**DOI:** 10.1038/s41598-024-62787-5

**Published:** 2024-07-14

**Authors:** Chiara Schiattarella, Alessandra Di Gaspare, Leonardo Viti, M. Alejandro Justo Guerrero, Lianhe H. Li, Mohammed Salih, A. Giles Davies, Edmund H. Linfield, Jincan Zhang, Hamideh Ramezani, Andrea C. Ferrari, Miriam S. Vitiello

**Affiliations:** 1 NEST, CNR-NANO and Scuola Normale Superiore, 56127 Pisa, Italy; 2 School of Electronic and Electrical Engineering, University of Leeds, Leeds, LS2 9JT UK; 3 Cambridge Graphene Centre, University of Cambridge, Cambridge, CB3 0FA UK

**Keywords:** Imaging and sensing, Nanoscale devices

## Abstract

Optical resonators are fundamental building blocks of photonic systems, enabling meta-surfaces, sensors, and transmission filters to be developed for a range of applications. Sub-wavelength size (< λ/10) resonators, including planar split-ring resonators, are at the forefront of research owing to their potential for light manipulation, sensing applications and for exploring fundamental light-matter coupling phenomena. Near-field microscopy has emerged as a valuable tool for mode imaging in sub-wavelength size terahertz (THz) frequency resonators, essential for emerging THz devices (e.g. negative index materials, magnetic mirrors, filters) and enhanced light-matter interaction phenomena. Here, we probe coherently the localized field supported by circular split ring resonators with single layer graphene (SLG) embedded in the resonator gap, by means of scattering-type scanning near-field optical microscopy (s-SNOM), using either a single-mode or a frequency comb THz quantum cascade laser (QCL), in a detectorless configuration, via self-mixing interferometry. We demonstrate deep sub-wavelength mapping of the field distribution associated with in-plane resonator modes resolving both amplitude and phase of the supported modes, and unveiling resonant electric field enhancement in SLG, key for high harmonic generation.

## Introduction

Metamaterials or metasurfaces, consisting of miniaturized optical resonators with subwavelength periodicity, can manipulate the wavefront of incident waves, shaping wavefronts either through reflection or transmission^1^. In the terahertz (THz) frequency range, defined between 0.1 THz and 10 THz, high-efficiency resonators (field enhancement > 10^4^)^2^ are particularly important^3,4^. They can be integrated into THz modulators to reduce the footprint and improve device efficiency^5^, be adapted to compact (< 0.5 mm^3^)^6^ THz sources to engineer their emission, hence reducing power consumption^7^ and manipulating spectral properties (e.g. frequency, beam shaping)^8^, or adopted as individual elements of fast (speed ~ 100 MHz) receivers^9^.

Applications of sub-wavelength metallic resonators span sensing^10,11^, filtering^12–14^, wavefront manipulation^9^, single micro-organisms detection exploiting highly localized (< λ/100) THz fields^15^, and investigation of strong light-matter coupling (normalized coupling ratios > 0.8)^16^ in quantum heterostructures^17^.

Many of the metamaterials developed so far in the far-infrared are based on noble metals, taking advantage of their negative permittivity below the plasma frequency^18^. Amongst them, split ring resonators (SRRs), originally proposed to achieve a controllable magnetic susceptibility^19^ have been largely adopted in the THz for cavity-enhanced light–matter interactions in semiconductor heterostructures^20^, enabling efficient concentration and enhancement of the electric field^21,22^.

However, the ability to tune a metamaterial is a fundamental requirement, especially in applications such as optical switches and modulators, but this is a property lacking in metals due to their intrinsic optical losses^23^. The emergence of layered materials (LMs)^24^, such as graphene, with its tunable carrier density^25^, large carrier mobility (> 10^4^ cm^2^/Vs at room temperature)^26^, ease of integration and compatibility with semiconductor substrates^27^ enabled the realization of tunable metamaterials in the THz frequency range^28,29^. Graphene can support tightly confined (10^6^ times < diffraction limit)^30^ THz surface plasmons (SPs)^31,32^, it can be patterned into various shapes including meta-atoms^33^ or closely packed ribbons^34^, and can be easily embedded in metamaterials and resonators^35^. The subwavelength nature of the resulting tunable metamaterial can enable enhanced light–matter interaction^36–41^ but, at the same time, the mode confinement poses a challenge for the experimental characterization of its properties. A possible approach to overcome this issue relies on probing metamaterial arrays in the far-field, with the inherent disadvantage of collecting spurious signals arising from the mutual radiative, plasmonic or capacitive coupling between individual elements^42^. Hence, near-field investigations are best suited to fully capture the interaction of THz waves with metamaterials and sub-wavelength resonators. Previous near-field approaches relied on the use of miniaturized photoconductive or electro-optic probes^43–46^, or aperture-type near-field probes of micrometric dimensions^47^; however, the latter typically suffer from a spatial resolution constrained to ∼ 1–30 μm and a limited dynamic range^47^.

Scattering-type near-field optical microscopy (s-SNOM) and aperture-type near-field microscopy (a-SNOM) can overcome the limitations of far-field approaches and probe the nanoscale properties of metamaterials^48^. In particular, THz s-SNOM enables extremely sub-wavelength resolutions (< λ/1000)^49^ for the reconstruction of light-matter interaction effects in the real space^50^, capturing collective propagating modes such as Dirac hybrid plasmon polaritons in LM-based metamaterials^51^ or the modal distributions of individual metamaterial resonators^52–55^.

Here, we characterize individual metallic circular split ring resonators (CSRRs), with single layer graphene (SLG) embedded in the resonator gap, via s-SNOM, in a detector-less, amplitude and phase-sensitive configuration, making use of a quantum cascade laser (QCL) as a source and as a detector, through the self-mixing effect^56^. We capture the electric field confinement and enhancement provided by the CSRR in SLG, mapping the resonator modes under different polarizations of the incident THz beam. Furthermore, we perform hyperspectral nano-imaging employing a multi-mode frequency comb (FC) source.

To limit the optical screening of the metallic CSRR array and the consequent resonance bleaching arising when the SLG film covers the entire ring area, we integrate it only in the CSRR split gap. The CSRR design offers the unique possibility to exploit the split gap for SLG embedding outside the metallic region, allowing the peak electric field to be concentrated in a portion of the active material where no screening or absorption from the metallic parts of the array dominates. This allows to retrieve the near-field enhancement at multiple frequencies and takes full advantage of the nonlinearity of SLG, which is pivotal for pursuing nonlinear applications including high harmonic generation^57^.

## Results and discussion

**Figure 1:**
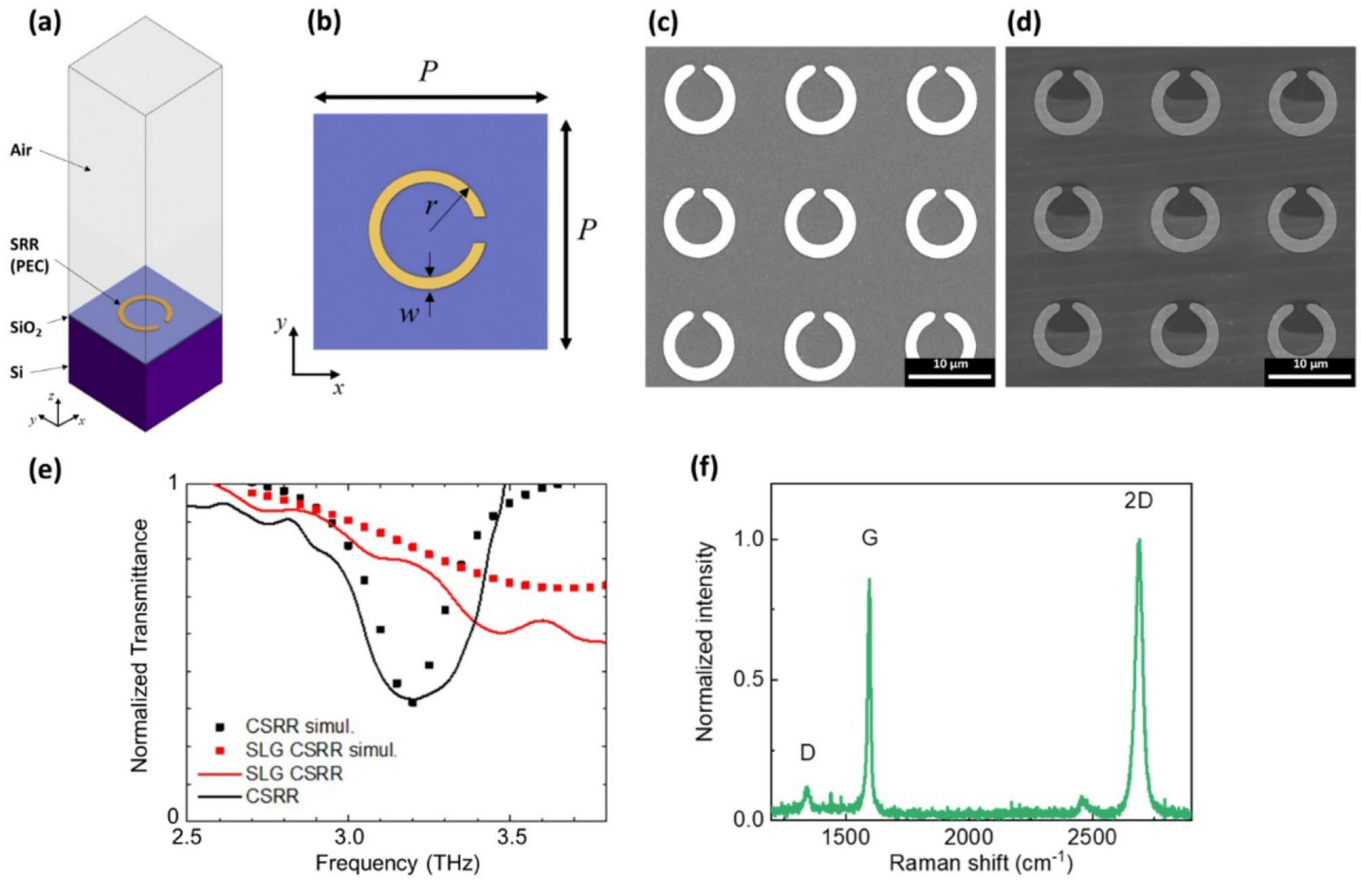
(**a**) Schematic of finite element three dimensional simulation design (PEC indicates a perfect electric conductor); (**b**) design of the unit cell of bare CSRR (P = pitch, r = outer radius, w = Au width); (**c**,**d**) SEM micrographs of as-fabricated CSRR and SLG-CSRR array, respectively; (**e**) normalized transmittance acquired via time domain spectroscopy of metallic CSRR with (red), and without (black) SLG in the gap. The corresponding simulated curves are reported for comparison as dotted curves. (**f**) Raman spectrum of SLG transferred on CSRR Au array and patterned in each split gap.

Individual CSRRs are designed to achieve a resonance at 3.2 THz via finite element method (FEM) simulations (Comsol Multiphysics) implementing the 3D model depicted in Fig. 1a (see “Methods”). The geometric parameters of the unit cell (ring radius, Au width, gap size, pitch) are optimized (Fig. 1b) to increase the CSRR electric field enhancement, defined as the ratio between the average field in the resonator gap and that in an equally extended surface outside the gap region, i.e. at the ring center. Scanning electron microscopy (SEM) images of the corresponding device, before and after SLG integration, are in Fig. 1c,d.

The SLG-CSRR transmittance, measured by time domain spectroscopy (TDS) under dry air purging (Fig. 1e, details in “Methods”), before and after SLG integration (SLG-CSRR), confirms the occurrence of a resonance around the desired frequency, *f*_CSRR_ = 3.20 THz. The absorption appears blue-shifted to *f*_SLG-CSRR_ = 3.36 THz after SLG integration. At the same time, a decrease of the experimental quality factor from the pristine *Q*_CSRR_ = *f*_CSRR_/Δ*f*_CSRR_ = 11 to *Q*_SLG-CSRR_ = 2.3 is observed, as expected by simulations. SLG intraband conductivity is a doping- and scattering time-dependent function^58^:

1 $${\sigma }_{intra}\left(\nu \right)=\frac{-i{D}_{0}}{\pi }\frac{1}{\left(2\pi \nu +i{\Gamma }_{0}\right)}$$

where ${D}_{0}={E}_{F}{e}^{2}/{\hbar }^{2}$ is the linear Drude weight, *e* is the electron charge, *ħ* is the reduced Planck constant, ${\Gamma }_{0}={\tau }_{0}^{-1}=e{{v}_{F}}^{2}/{E}_{F}\mu$ is the scattering rate, *E*_F_ the Fermi energy, *v*_F_ the Fermi velocity, and *µ* is the carrier mobility. The SLG *E*_F_ is extrapolated via Raman spectroscopy (Fig. 1f)^57^. The micro-Raman spectrum of as-grown SLG on Cu comprises the characteristic G and 2D peaks^59^ (details in Supporting Information, Figure S1). The post processing quality of the employed graphene has been checked via micro-Raman spectroscopy as well. By probing four different regions on the sample surface, we get Pos(G) = 1594 ± 6 cm^−1^, Pos(2D) = 2675 ± 4 cm^−1^. The occurrence of a weak D peak, with I(D)/I(G) = 0.086 ± 0.002, implies the presence of Raman-active defects^60,61^, ascribable to the limited size of the embedded SLG flake at the end of the processing. *E*_F_ = 250 meV is estimated from I(2D)/I(G) = 1.98 ± 0.7, A(2D)/A(G) = 3.8 ± 0.7^62,63^. The scattering time *t*_0_ = 23.4 fs is extracted from the estimated SLG mobility *µ* = 1100 cm^2^/Vs^64^. We then perform FEM simulations, implementing two ports, one transmitting and the other receiving the plane wave impinging on the CSRR array unit cell, to simulate the frequency-dependent transmittance, center frequency (ν_0_) and quality factor (*Q*), whose extracted resonance values (through a Voigt fit) well reproduce the experimental blue-shift and broadening of the absorption dip. Specifically, ${\nu }_{0}^{(CSRR)}$ = 3.19 THz, ${Q}_{CSRR}^{(simul)}$ = 11.8, ${\nu }_{0}^{(SLG-CSRR)}$ = 3.36 THz and ${Q}_{SLG-CSRR}^{(simul)}$ = 2.4, respectively.

**Figure 2:**
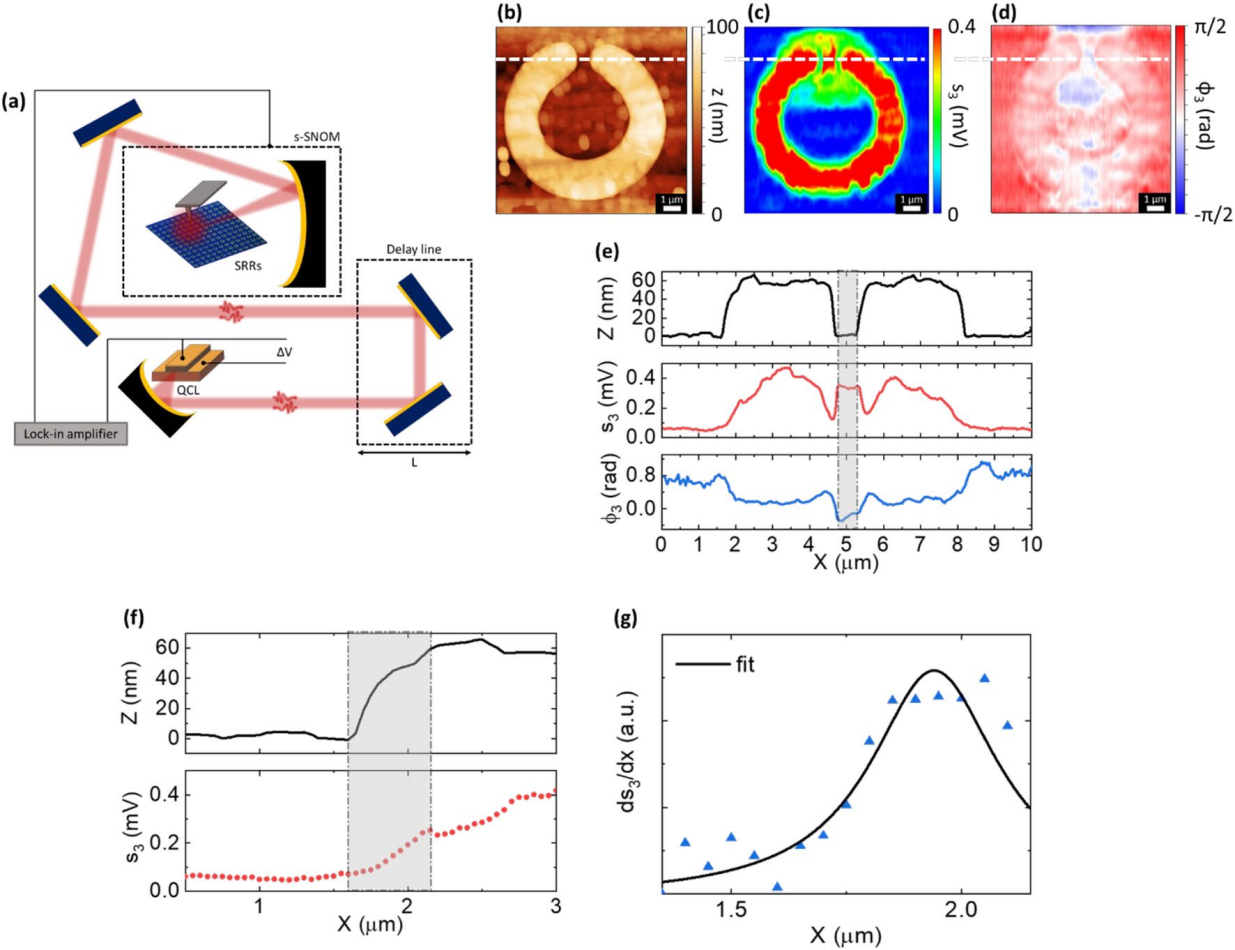
(**a**) Schematic of near-field detector-less s-SNOM setup. (**b**) Topography *z*, (**c**) reconstructed near-field amplitude *s*_3_ and, (**d**) phase *φ*_3_ images of a single CSRR with SLG embedded in the gap. The self-mixing (SM) signal is demodulated at the 3rd harmonic of the tapping frequency, and the pumping source is a QCL operating at *f*_QCL_ = 3.0 THz (current I_QCL_ = 370 mA, temperature T_QCL_ = 8.8 K). (**e**) Line-cut profiles of *z*, *s*_3_ and *φ*_3_ extracted along the white line marked in panels (**b**–d). (**f**) Evaluation of the spatial resolution of the THz s-SNOM experiment. (**g**) Lorentzian fit for the line response function (LRF) correspondent to the grey-shaded X interval in (**f**).

The SLG-integrated CSRR is then investigated in the near-field by using the experimental set-up sketched in Fig. 2a. Rather than the well-established pseudoheterodyne scheme^65,66^, we employ a detector-less technique, exploiting the self-mixing (SM) phenomenon in the cavity of a QCL pumping source, occurring when back-scattered photons are re-injected in the QCL cavity itself^67^. This translates into a change of the QCL voltage that serves as a transduction quantity (Fig. 2a)^67,68^. In addition to the inherent advantages of QCL technology in terms of compactness, narrowband excitation and spectral coverage over the range ∼1.2–5.5 THz^69^ and tens mW output powers in continuous wave (CW)^70^, the SM scheme offers low noise-equivalent powers (∼pW/√Hz)^71^, hence ideal to detect weak fields back-scattered from the THz s-SNOM tip^72^.

We initially employ a single plasmon waveguide, TM-polarized, single-frequency QCL operating at *f* = 3.0 THz (driving current *I*_QCL_ = 370 mA, heat sink temperature *T*_QCL_ = 8.8 K) and demodulate the back-scattered signal at higher harmonics (herein the 3rd one) of the probe tip tapping frequency to ensure background-free data^48^.

To retrieve the real-space distribution of the near-field SM signal amplitude s_3_ and phase ϕ_3_ from s-SNOM images, we employ synthetic optical holography (SOH)^73^. The data acquisition is simultaneous to the signal phase modulation, achieved by regulating the overall optical path with a delay line. This enables the collection of interferometric patterns that can be analyzed via discrete Fourier transform (DFT) to retrieve the spectral content of the SM fringes, which intrinsically contain information on the emission spectrum of the source^51^.

Figure 2b–d show the topography and the reconstructed 3rd order near-field amplitude and phase of an individual SLG-CSRR. The holographic maps are collected by scanning 220 × 500 pixel images (11 × 11 µm^2^) while moving the delay line in Δ*L* = 5 μm steps at each scanning line along the *Y* direction. The difference in the pixel size along the *X* and *Y* directions is set to achieve a sufficient SM fringe sampling, so to ensure a suitable spectral resolution (< Δν_QCL_), while retaining the spatial information. DFT analysis of the resulting holograms is then performed to reconstruct the amplitude and phase maps of the backscattered near-field signal.

The near-field map of the third-order self-mixing amplitude signal (s_3_, Fig. 2c) has an optical contrast modulation that reveals the different reflectivity of the SLG-CSRR array elements: metal ring, SLG film and Si/SiO_2_ substrate. The near-field signal from the SLG surface outside the gap region is estimated to be ~ 3 times larger than the corresponding signal from the insulating Si/SiO_2_. This is expected due to the higher optical contrast/local reflectivity of SLG when compared to Si/SiO_2_ in the THz range^74^. Most importantly, an enhancement ~ 2 of SLG near-field signal is observed in the resonator split gap, Fig. 2c.

To estimate the spatial resolution Δx of our THz s-SNOM experiment, we evaluate the line response function (LRF) as conventionally performed in microscopy techniques^75,76^. To this end, the first-order spatial derivative of the *s*_3_ profile corresponding to the substrate/Au interface (referred to as edge response function, ERF) is fitted to a Lorentzian peak, extracting a full width at half-maximum FWHM (~ Δx) = 340 ± 100 nm, corresponding to ~ λ/300 (Fig. 2f,g).

For a TM-polarized incident beam, the interaction with the probe tip is favored with respect to TE-polarized radiation^77^. This implies that out-of-plane modes sustained by a resonating structure are more likely to be scattered to the far-field, whereas in-plane field components poorly interact with the vertically aligned probe. The TM-polarized scattered field can be written as:

2 $$E_{{{\text{scat}}}} = \alpha_{{\text{d}}} E_{{{\text{orth}}}} + \alpha_{{\text{z}}} E_{{\text{z}}} ,$$

with *E*_orth_ being the out-of-plane component of the incident field, *α*_d_ the complex scattering efficiency that includes the near-field tip-sample dipolar interaction^65^, *E*_z_ represents the out-of-plane field component associated to the resonant modes of the metasurface and *α*_z_ quantifies its scattering efficiency.

In the regime of weak optical feedback (i.e., in the sinusoidal-shaped interferometric SM fringes case^68^), adopted in the present case, the SM voltage signal is proportional to the cavity-reinjected back-scattered field, apart from an accumulated roundtrip phase *ϕ*_ext_ = 4π*L*_ext_*f*/*c*, with *L*_ext_ the total distance between QCL facet and probe tip^78^. Hence, the SM signal can be expressed, in terms of amplitudes and phases, as a sum of individual contributions corresponding to the respective field terms:

3 $$\Delta V_{{{\text{SM}}}} = \, \left( {A_{{{\text{bulk}}}} {\text{e}}^{{ - {\text{i}}\phi {\text{bulk}}}} + A_{{\text{z}}} {\text{e}}^{{ - {\text{i}}\phi {\text{z}}}} } \right){\text{e}}^{{{\text{i}}\phi {\text{ext}}}}$$

where *A*_bulk_ contains the variations in the local permittivity of the sample and *A*_z_ is insensitive to bulk properties and solely provides information on the spatial distribution of the out-of-plane resonant modes supported by the sample^53^.

**Figure 3:**
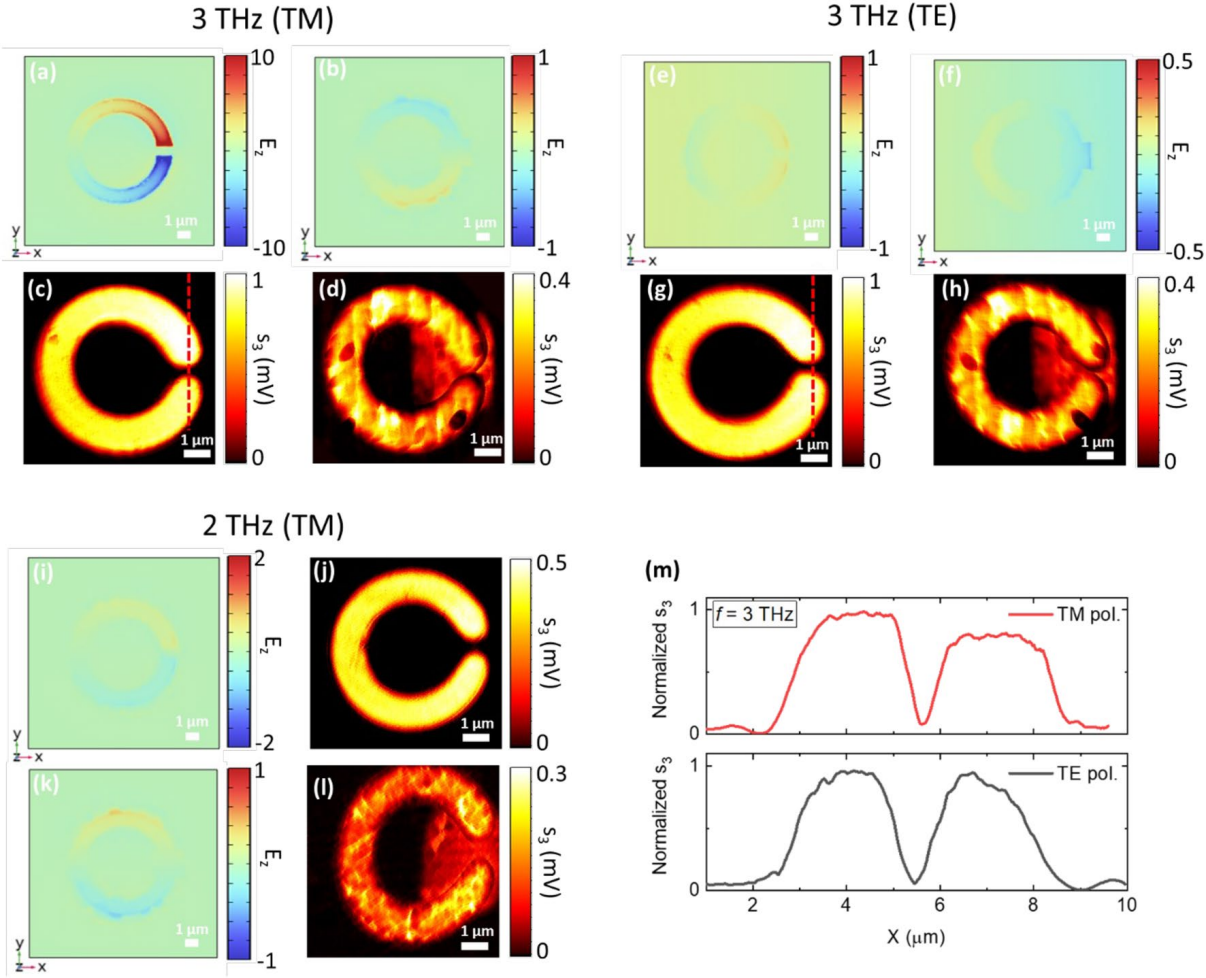
Simulated (top) and experimental (bottom) near-field amplitude distributions of individual metallic CSRR with and without SLG in the gap: (**a**–**d**) bare and SLG-embedded *on resonance* CSRR (incident frequency 3.0 THz, TM polarization); (**e**–**h**) bare and SLG-embedded CSRR (incident frequency 3.0 THz, TE polarization); (**i**–**l**) bare and SLG-embedded CSRR in *off-resonance* conditions (incident frequency 2.0 THz, TE polarization). (**m**) Line-cut profiles of experimental near-field distribution of bare CSRRs along the red dashed lines depicted in (**c**,**g**). The field enhancement trace is visible when tuning on-resonance (**a**).

To visualize the modes driven by the incoming THz wave, impinging at an incidence angle of 54° according to the experimental optical path dictated by the s-SNOM microscope, the magnitude of the out-of-plane electric field component *E*_z_ is evaluated in the *X–Y* plane, 50 nm above the CSRR surface, via FEM simulations (see “Methods”), then compared with the experimental images of bare and SLG-embedded CSRRs (Fig. 3). Herein the 3rd order demodulated signal is considered. At an incident frequency *f*_QCL_ = 3 THz and under TM polarization (Fig. 3a–d), a symmetric field localization could be evidenced from the bare CSRR simulations. SLG-embedded CSRRs exhibit unavoidable impurities due to the fabrication process that prevents a clear sorting of the resonance-driven surface current modes, which represent only a small contribution to the total signal. The main way to reveal a resonant phenomenon is the field enhancement trace of SLG within the split gap region, as shown in Fig. 2 and as discussed below.

To confirm that the field enhancement is a resonant effect, we repeat the experiment using as pump source a single-frequency QCL emitting at a detuned frequency *f*_QCL_ = 2.0 THz (I_QCL_ = 675 mA, T_QCL_ = 6.8 K). The same measurements were also repeated at 3.0 THz by rotating the sample at 90° to mimic incident TE-polarization and, therefore, to further sort out the contributions of the two terms in the SM signal. At incident TE polarization (Fig. 3e–h) and at *f*_QCL_ = 2.0 THz (Fig. 3i–l), no field localization is detected. Concurrently, the near-field trace of graphene is evidenced due to its THz reflectivity, comparable to that of the Au films in the same frequency range^74^. Off-resonance, the image contrast is only dependent on the local variations of the dielectric function in the near-field back-scattered signal from the s-SNOM tip: *A*_bulk_. This further highlights the resonant mode of the CSRR at 3 THz and TM polarization, since it is reflected in an asymmetry in the experimental near-field distribution along the two arms of the ring. Such behavior is absent in the incident TE-polarization case (Fig. 3m).

**Figure 4:**
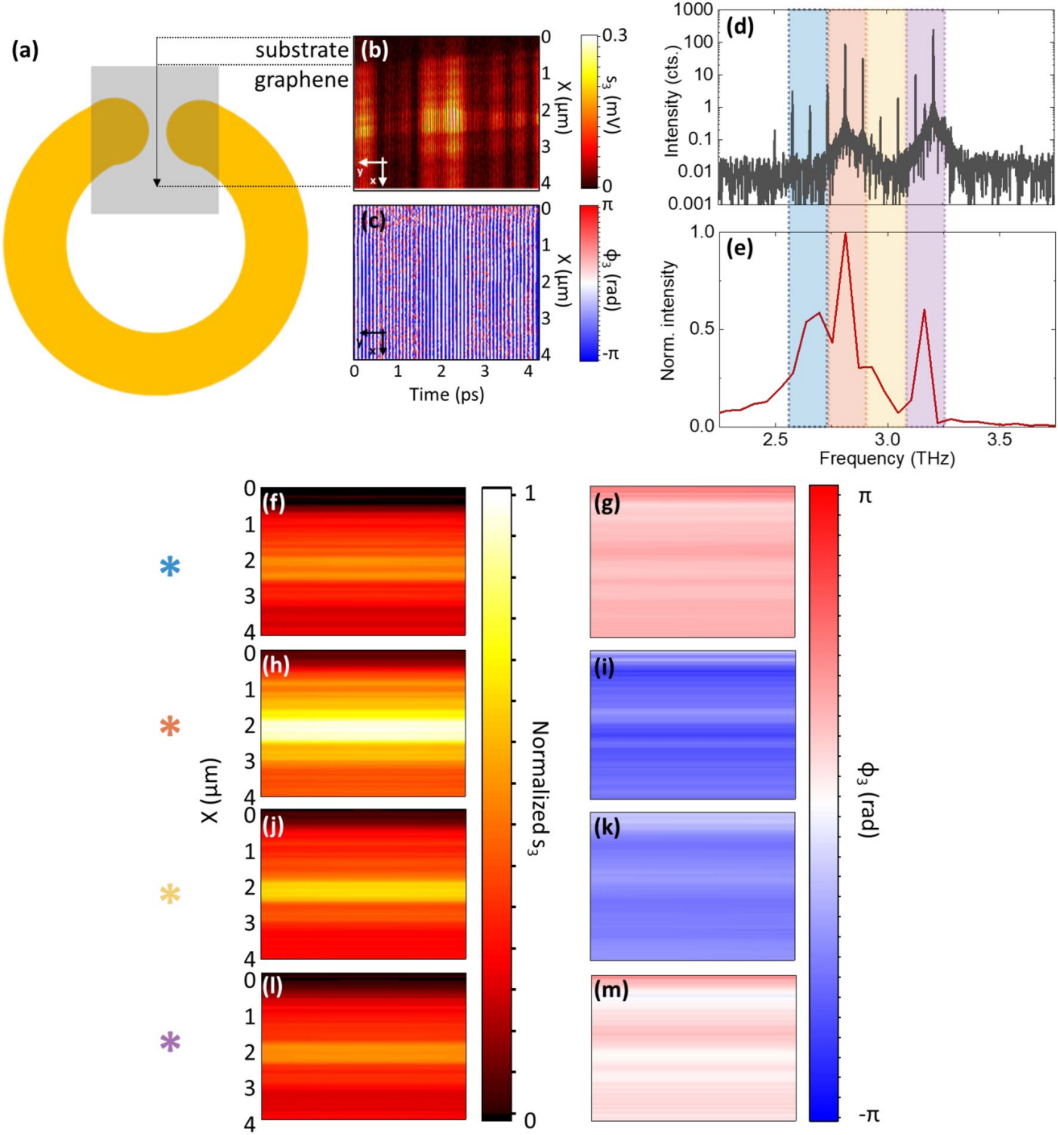
(**a**) Schematic of the scanned region (SLG within CSRR gap). (**b**,**c**) Near-field hologram (amplitude *s*_3_ and phase* ϕ*_*3*_) of the 0.3 µm wide substrate/SLG strip along the gap of the CSRR. (**d**) FTIR emission spectrum of pump source, a QCL FC driven at I_FC_ = 620 mA; (**e**) power spectrum of FC after Fourier transform of the interferometric patterns reported in (**a**,**b**). The colored areas identify the spectral components ~ 2.65, 2.83, 2.93 and 3.18 THz obtained from the convolutions of the peaks in (**d**), reflected in the near field images; this probes the CSRR response within a frequency span of 530 GHz. (**f**–**m**) Reconstructed two-dimensional amplitude *s*_3_ and phase *ϕ*_3_ maps of the selected spatial region at the four aforementioned frequencies, acquired at I_FC_ = 624 mA and at heat sink temperature T_FC_ = 26.5 K.

We then perform nano-imaging in the near-field, using a multifrequency pump beam, following the same procedure adopted for the single-frequency QCL. We scan, over a 4 × 0.3 µm^2^ area, a SLG strip along the gap of the single CSRR, as for Fig. 4a. While the SLG-CSSR is scanned in position, the optical path length *L*_ext_ is varied to modulate the phase of the reference field, spanning the time interval to optimize the sampling required to achieve a suitable spectral resolution, according to the Nyquist theorem^79^. This approach allows to reconstruct a bi-dimensional hologram (Fig. 4b,c).

A THz QCL frequency comb (FC), whose FT-IR emission spectrum is shown in Fig. 4d, close to the designed resonant frequency of the CSRR, is adopted as source and detector, simultaneously. FCs provide superior sensitivity to optical feedback owing to their phase-locked operating regime^80^ and the acquired amplitude and phase interferometric patterns intrinsically encode information on the optical response of the sample at the multiple emitted frequencies.

Figure 4b,c plot the third-order near-field SM amplitudes and phase maps acquired on the previously mentioned sample region. The amplitude (Fig. 4b) exhibits periodic oscillations induced by the phase modulation along the *y* direction that results from the beating of the different modes that contribute to the SM signal of the frequency comb. The spectrum of the third harmonic near-field SM signal ${\sigma }_{3}$= *s*_3_ exp(iϕ_3_) (Fig. 4e), while driving the FC at I_FC_ = 624 mA and at T_FC_ = 26 K, shows that, despite only part of the comb modes contribute to the self-detected near-field signal (Fig. 4d)^80^, an optical bandwidth ~ 530 GHz is spanned. Four main spectral components can be associated with the convolution of the most intense modes marked by the colored areas in Fig. 4e.

The amplitude and phase maps are analyzed by DFT to retrieve the real-space distribution of the local field corresponding to the four detected spectral components (Fig. 4f,h,j,l for amplitude, Fig. 4g,i,k,m for phase) of the FC (Fig. 4e).

**Figure 5:**
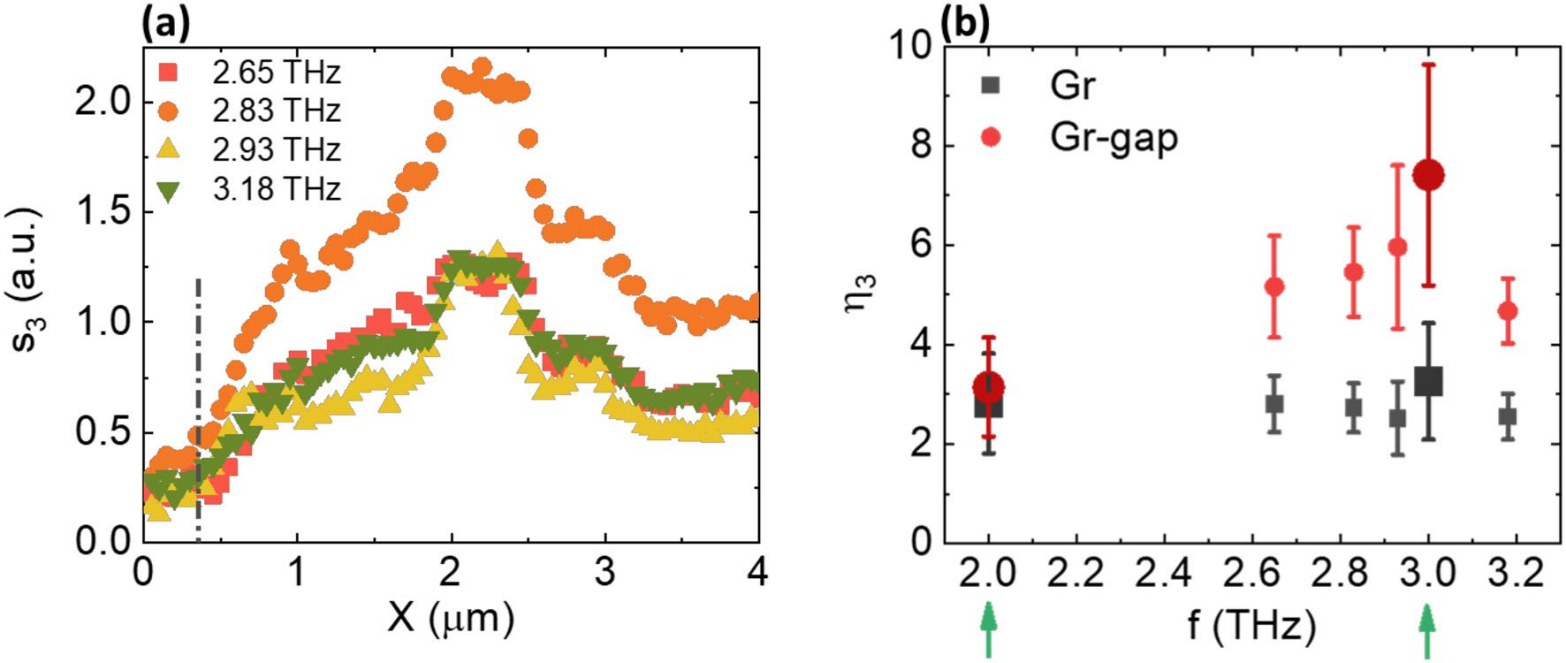
(**a**) Averaged profiles of *s*_3_ of the four FC frequencies along the X direction as retrieved from the power spectrum in Fig. 4e. The black dash-dotted line denotes the substrate/SLG interface as noticeable from Fig. 4b,c. (**b**) Evaluation of the optical contrast *η*_3_ as a function of probe frequency within and outside the CSRR gap. Data extracted from both FC and single-frequency QCL measurements (the latter marked with the green arrows/bigger data points) are shown.

A portion of the SiO_2_ substrate was purposely imaged to investigate the SLG/substrate interface. Figure 4c,e,g,i indicate a visible electric field concentration in the CSRR gap, that persists for all FC frequencies. The observed enhancement of the localized field is quantified by estimating the near-field contrast *η*_3_ = *s*_3_(SLG)/*s*_3_(SiO_2_) when probing the proximity of the CSRR hotspot. An improvement in *η*_3_ by a factor ~ 2 was estimated, consistent with the experimental *Q*-factor of the SLG-CSRR resonance, with fluctuations between the different frequencies. The averaged spatial profiles of *s*_3_ at the four FC frequencies and the corresponding values of *η*_3_ are reported in Fig. 5a,b, respectively. Data referring to the single-frequency characterizations at 2 and 3 THz are also shown, confirming the overall trend.

In proximity of the SLG in the CSRR gap, a field enhancement is retrieved for all FC modes, even when their frequency is detuned from the nominal resonance (Fig. 1b). The far-field can only capture the average behavior of the CSRR array and, in general, of any metasurface system, evidencing spectral broadening effects^42^. Frequency shifts between near- and far-field signals were previously reported in dipolar plasmonic nanoantennas^81^.

Finally, it is worth noting that in s-SNOM imaging, THz radiation is focused onto the sample at a specific angle of incidence (see “Methods”), rather than being collimated along the normal direction. This implies that, in reciprocal space, a finite interval of wavevectors excites the system, i.e., a specific area of the Brillouin zone associated to the single metaelement is probed. Since the TM dispersion of CSRR metasurfaces is not trivial^82^, this angular spread may lead to a frequency detuning of the resonance.

## Conclusion

In this study, we combined time domain spectroscopy and detector-less near-field nanoscopy, at a single-frequency and in a multiwavelength detectorless configuration, to investigate the far- and near-field responses of a SLG-integrated CSRR array. The optical response in the far-field unveils an absorption dip at ~ 3.2 THz, in agreement with the designed resonance. THz s-SNOM enables spectroscopic investigations of resonant modes supported by individual metallic CSSRs avoiding inter-resonator coupling effects present in the far-field characterization of metasurface arrays. The supported resonant modes are traced in- and out- of resonance and under cross-polarization measurement with deep sub-wavelength spatial resolution (~ λ/300). By probing the meta-element with a THz QCL FC, we experienced a visible enhancement of the near-field signal within SLG, consistent with the experimental quality factor *Q* of the designed CSRR. This effect is absent when exciting the system spectrally far off-resonance. Simultaneously, the observation of a resonant field concentration in proximity of the SRR gap, at slightly detuned conditions, could be ascribed to the concurring action of a finite interval of incident wavevectors, since the THz radiation is focused onto the system and not collimated, and to the concurrent interaction between neighboring meta-elements, factors that cause a shift and a broadening of the resonance. Our results pave the way towards to the development of tunable optical circuits that exploits light-matter interaction phenomena at the nanoscale.

## Methods

### Fabrication of SLG-CSRRs

SLG is grown on Cu foils (35 μm thick) at 1050 °C via low-pressure chemical vapor deposition (CVD), employing a quartz tube furnace. SLG on Cu is transferred onto the target substrates via wet transfer^83^: A4-950 K ply(methyl-methacrylate) polymer (PMMA) is spin coated at 2000 rpm on the surface of the sample (1 cm^2^), followed by 1 min baking on a hot plate at 90 °C. Mild oxygen plasma treatment is utilized to remove SLG on the other side. The PMMA/SLG/Cu sample is then placed in a solution of 1 g of ammonium persulfate and 40 ml of deionized (DI) water to etch the Cu foil. Once Cu etching is complete, the PMMA-SLG film is transferred in DI water. This is left to dry overnight and finally the PMMA is removed with acetone.

A set of CSRRs is fabricated in an array configuration by electron beam lithography on a high (10^4^ Ω cm)-resistivity 300-mm-thick Si substrate coated with 300 nm SiO_2_ (by Siltronyx). The CSRR array pattern is defined by optical lithography using a LOR3A/S1805 bilayer photoresist, on a 6 × 6 mm^2^ area, followed by metal evaporation and liftoff of 10 nm/80 nm of Cr/Au. SLG is then transferred on the CSRR array, via a PMMA-assisted method^83^. A second step of optical lithography is then performed to define the SLG in the gap area, followed by a plasma-O_2_ etching to remove the SLG film from the desired area, and a final cleaning by acetone soaking.

### Time domain spectroscopy

The (SLG-)CSRR transmittance is measured by time domain spectroscopy under purged atmosphere (Menlo Terasmart k5), with a delayed-pulse sampling window of 82 ps, resulting in a spectral resolution ~ 15 GHz. The linearly polarized collimated beam spot size is 6 mm, i.e., comparable with the active area of the CSRR array. The sample is kept in a N_2_ purged environment (Water percentage > 3.5%) to suppress atmospheric absorption lines. The time-domain acquisition window is cutoff after the primary pulse detection, to avoid the back reflected pulse contribution to the transmittance curve. The transmittance curves are normalized by the reference sample trace, acquired on a bare SiO_2_/Si substrate belonging to the same batch used for the realization of the SLG-CSRR sample.

### CSRRs FEM simulations

A finite element method (FEM) is implemented with Comsol Multiphysics to derive the eigenvalue solutions of the Maxwell’s equations to find the resonance frequencies of our 3d model (Fig. 1a) as well as its frequency response. The model comprises a unit cell of the CSRR with an external radius (*r*) of 4.1 μm and 1 μm width (*w*), patterned on a SiO_2_/Si dielectric (300 nm/35 μm) defined by their refractive indices, 2.12 and 3.4, respectively. The gap width defining the CSRR is 1 μm. The volume on top is defined as air (refractive index n = 1), which represents the incidence medium. Since the CSRR (200 nm) is thicker than the penetration depth in the simulated frequency range, it is modeled as a perfect electric conductor. To retrieve the eigenfrequencies, all the external boundaries of the model are assigned as scattering boundaries. Conversely, for the harmonic propagation simulation, Floquet periodic boundary conditions^84^ are used on the four sides of the unit cell to simulate an infinite two-dimensional array. The top and bottom boundaries are set as port conditions, the top one represents the input port, and the bottom the output port. The port orientations to define the inward direction, the polarization state, and the incidence angle (54°) are specified at the input port. The scattering and port boundary conditions on the top and bottom boundaries allow the simulation of virtually infinite thick volumes, whereas the periodicity (*P*) of the unit cell is set as 15 μm. The simulations are performed in a frequency range between 1.5 and 4.2 THz under incident TE and TM polarizations.

### Near-field set-up

Near-field s-SNOM experiments are performed with a commercial NeaSNOM system by Neaspec/attocube (Attocube, Martinsried, Germany) employing a Pt-Ir probe tip (25PtIr300B-H40, radius 40 nm, resonance frequency 67 kHz, Rocky Mountain Nanotechnology) in tapping mode and keeping a tapping amplitude ~ 210 nm. THz QCLs at different frequencies (2.0 THz; 3.0 THz, and a frequency comb probed in the range [2.65 THz, 3.18 THz] are used as source/detector and mounted in a liquid He continuous-flow cryostat sealed via a polymethylpentene window. The heat sink temperatures and current operating ranges are kept fixed to maximize the phase stability of the SM signal. The emitted THz beam is collimated using a 90° off-axis parabolic mirror (OAP) with an effective focal length of 50 mm and guided to the optical port of the NeaSNOM. An additional broadband off axis parabolic (OAP) mirror then focuses the beam onto the AFM tip at an angle of incidence of 54° with respect to the CSRR plane normal. Such optical path exactly coincides with that of the radiation backscattered from the probe tip and re-injected in the QCL cavity. The average optical path length from the QCL front facet to the s-SNOM tip is ~ 60 cm. The near-field SM signal is retrieved by lock-in detection of the voltage modulation across the QCL terminals. The signal is pre-amplified using a low-noise amplifier (DL Instruments, mod. 1201) and demodulated up to the highest harmonic order of the tapping frequency allowed by the electronic board of the NeaSNOM system (n = 5). For collecting the two-dimensional holograms, an optical delay-line equipped with two 45°, 2″ plane mirrors are employed and mounted on a linear translation stage having 0.1 μm resolution (Physik Instrumente, stepper motor stage M403.62S), which varies the optical path *L* and controls the phase of the optical feedback on demand.

### Supplementary Information

Supplementary Information.

## Data Availability

Data presented in this study are available on request from the corresponding author.

## Code availability

The relevant computer codes supporting this study are available from the authors upon request.
