# Supplementary material for: Terahertz near-field microscopy of metallic circular split ring resonators with graphene in the gap
Source: Sci Rep. 2024 Jul 14;14:16227. doi: 10.1038/s41598-024-62787-5 (PMC11247082; doi:10.1038/s41598-024-62787-5)
Supplement: Supplementary file 1 — Supplementary Information. [file 41598_2024_62787_MOESM1_ESM.docx]

**Supplementary Information**

**Terahertz near-field microscopy of metallic circular split ring resonators with graphene in the gap**

Chiara Schiattarella,^1^ Alessandra Di Gaspare,^1^, Leonardo Viti,^1^ M. Alejandro Justo Guerrero,^1^ Lianhe H. Li,^2^ Mohammed Salih,^2^ A. Giles Davies,^2^ Edmund H. Linfield,^2^, Jincan Zhang,^3^ Hamideh Ramezani,^3^ Andrea C. Ferrari^3^ and Miriam S. Vitiello^1^

^1^NEST, CNR-NANO and Scuola Normale Superiore, 56127, Pisa, Italy

*^2^School of Electronic and Electrical Engineering, University of Leeds, Leeds, LS2 9JT, UK*

*^3^Cambridge Graphene Centre, University of Cambridge, Cambridge, CB3 0FA, UK*

**Raman spectroscopy**

Raman spectroscopy is performed on as-grown SLG/Cu at a 514 nm, using a 100× objective with an optical power density less than 0.5 mW/μm^2^ to prevent sample heating.

A typical Raman spectrum is shown in Fig. S1 after subtraction of the Cu photoluminescence.^81^ POS(G) =1585.5 ± 3.0 cm^-1^, POS(2D) = 2698.5 ± 3.3 cm^-1^. The 2D and G peaks are single Lorentzians with FWHM(2D) = 24.3 ± 2.8 cm^-1^, and FWHM(G) = 15.1 ± 2.0 cm^-1^. I(2D)/I(G) = 4.41 ± 0.99 and A(2D)/A(G) = 7.06 ± 1.25. No D peak is observed, indicating negligible defects. We derive E_F_= 228 ± 15 meV from Pos(G), A(2D)/A(G), I(2D)/I(G) and FWHM(G).^59,82,83^

The Raman spectrum of SLG after transfer and patterning on the split gap area of the CSRR (Fig. 1f of the Main text) is acquired with a Horiba (Xplora plus) at excitation wavelength 532 nm, focusing with a 100× objective to achieve a lateral resolution of ~2 µm, and keeping an incident optical power density of 0.4 mW/μm^2^ to prevent undesired sample heating.


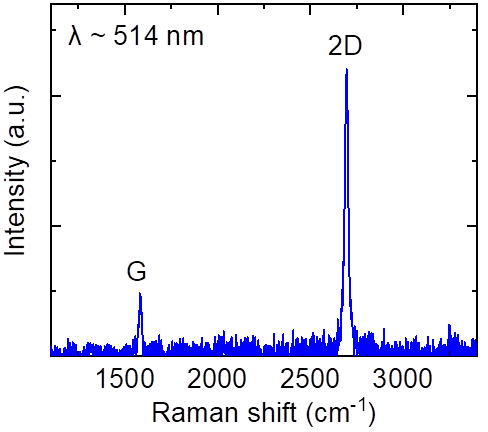


**Figure S1:** Raman spectrum of SLG on Cu, acquired with a 514 nm laser line.
